# An Unusual Case of May-Thurner Syndrome in a Middle-Aged IV Drug Abuser

**DOI:** 10.7759/cureus.29360

**Published:** 2022-09-20

**Authors:** Ali Danish, Adil S Mohammed, Sai Gautham Kanagala, Majid Aized, Ahmad Ali Khan

**Affiliations:** 1 Internal Medicine, Nishtar Medical University Hospital, Multan, PAK; 2 Internal Medicine, Mamata Medical College, Khammam, IND; 3 Internal Medicine, Osmania Medical College, Hyderabad, IND; 4 Vascular Surgery, Vascular Health Clinic, Midland, USA

**Keywords:** may thurner syndrome (mts), ivc filter, tpa treatment, ct venogram, deep venous thrombosis (dvt), oral anticoagulation, intravascular ultrasound (ivus)

## Abstract

May-Thurner syndrome (MTS) is an extrinsic venous compression of the iliocaval venous territory by the arterial system. MTS is common in middle-aged women. Despite its importance, it is uncommonly considered in the differential diagnosis of deep vein thrombosis (DVT), especially in males with other risk factors. Due to the perianal abscess, a 35-year-old male health care worker was abusing IV opioids through his left leg veins. His symptoms included signs and symptoms of cellulitis around the catheter site, followed by recurrent DVTs due to poor response to anticoagulation therapy alone. A comprehensive workup revealed the diagnosis of MTS. The patient eventually required endovenous treatment with stent placement, after which his condition improved dramatically.

## Introduction

May-Thurner syndrome (MTS) is a condition where the venous system is pushed against bony structures in the Iliocaval territory by the overlying arterial system. It can be asymptomatic or progress to symptomatic deep vein thrombosis (DVT). Symptoms of MTS include pain and swelling of the lower extremity, venous claudication, or chronic development of symptoms or signs of venous insufficiency. Cross-sectional imaging such as CT/MR venography is sensitive whereas Catheter-based contrast venography may need to be performed in anteroposterior and oblique angles to confirm the diagnosis. Obtaining two or three projections during the injection phase is important to improve accuracy [1]. Here, we present a case of MTS in a middle-aged IV drug abuser who developed left leg swelling and was treated for thrombosis with full therapeutic anticoagulation. Along with this, an IVC filter was placed to prevent the thromboembolic phenomenon which was followed by catheter-directed thrombolysis. Stenting of the iliocaval segment was also done [2].

## Case presentation

A 35-year-old man presented to the clinic with pain, swelling, and heaviness in his left leg. He reported being on IV antibiotics and opioids for 21 days due to a sizable perianal abscess. He also said that he continued to administer opioids for a further three weeks after his antibiotic course, having developed an addiction to overcome the pain associated with the abscess. The patient had an intravenous access catheter on the left dorsum of the foot, which was associated with extensive cellulitis. The patient is a health care worker who had easy access to a hospital pharmacy and used these medications for his personal use at home. He placed peripheral IV lines several times on both his arms which clogged up eventually leading to the placement of an IV line on his left foot.

On examination, the patient's weight was 90 kg, height was 170 cm, and BMI was 31 kg/m^2^. His vital signs were as below: blood pressure of 130/90 mm Hg; pulse rate of 88 beats per minute; respiratory rate of 18 per minute; and temperature of 98.6 F. Local examination revealed pitting edema of the left leg at and below the level of knee and some redness around the IV catheter. The perianal abscess had recovered. Another physical examination was unremarkable.

Firstly, the IV catheter was removed, and the patient was started on oral antibiotics and advised a Doppler venous study of the left leg, which revealed a distended left popliteal vein signifying thrombosis. He was started on low molecular weight heparin and afterward bridged to apixaban, leg elevation, and oral opioids for pain relief were advised.

**Figure 1:**
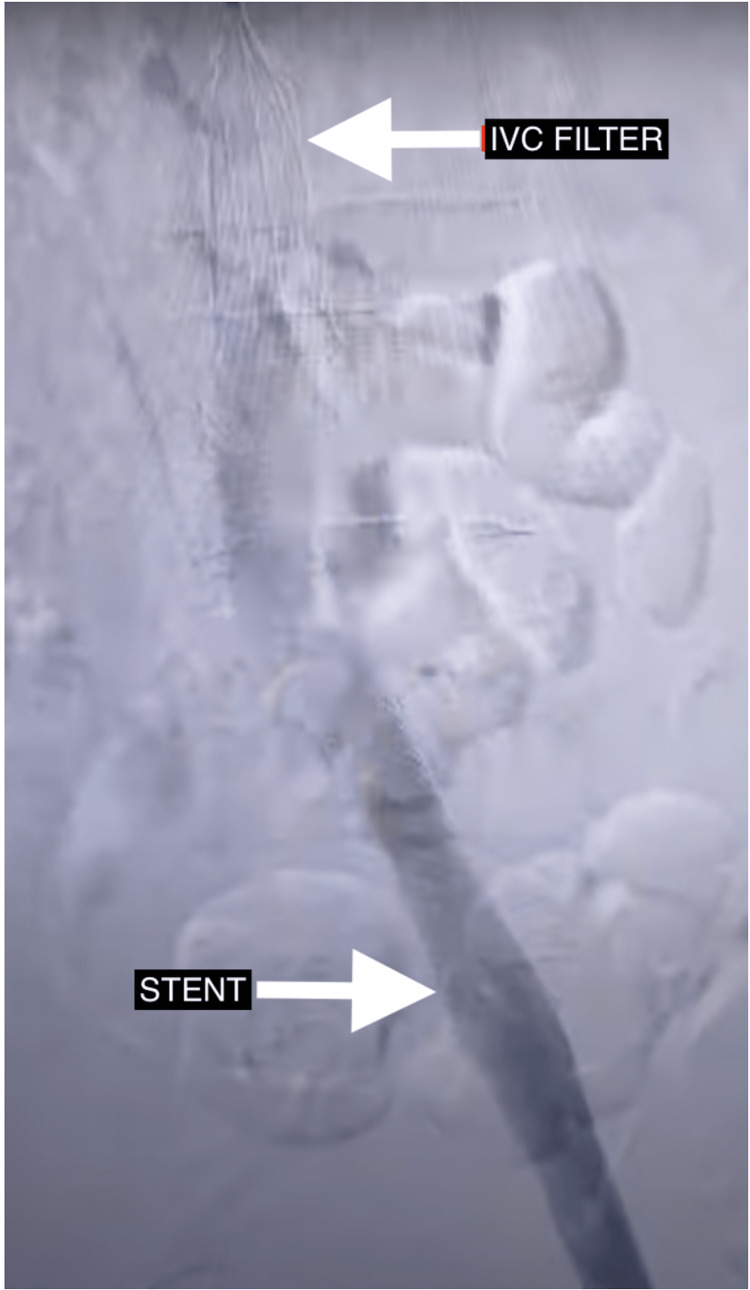
IVC filter in place to prevent dislodged blood clots from reaching the pulmonary circulation and stent in place to correct the narrowing of left iliac vein

A month later, the patient experienced increasing swelling and pain up to the level of the upper thigh and also had a couple of episodes of hemoptysis. He was compliant with his medications. CT chest, abdomen, and pelvis venogram revealed dilatation in the iliac veins associated with DVT extending into his iliac veins. Besides, there was tight stenosis of the left iliac vein at the junction, passing beneath the right iliac artery (Video 1) and showering emboli in the right pulmonary artery. Immediate treatment was begun with the placement of an inferior vena cava filter to prevent pulmonary embolism (with a plan to remove it in three months), catheter-directed tissue plasminogen activator was then injected into the deep veins of the thigh via popliteal fossa, followed by a drug-eluting balloon-expandable metal stent placement at the point of venous narrowing of the iliac vein to prevent the occlusion caused by overlying right iliac artery (Figure 1).

**Video 1:** Filling defect in proximal left iliac vein indicating thrombus formation distally to the point of occlusion

After the procedure, the swelling subsided dramatically. The patient continues to receive apixaban anticoagulation therapy and had a monthly check-up. A thrombophilia panel workup was also performed in this patient which was normal.

## Discussion

MTS is a rare disorder that presents with compression of a left iliac vein due to the overlying right iliac artery which causes thrombosis of the deep leg veins. Symptoms include leg swelling, associated pain, and fullness. DVT of the proximal veins of the leg is the most common presentation. There have been a few predisposing factors for this disease like pregnancy, prothrombotic state, IV drug abuse, prolonged immobilization, post-surgery, or after taking contraceptive pills [3]. This disorder is also referred to as Cockett or left iliac vein compression syndrome. MTS is known to occur mostly during the second to fourth decades of life, predominantly in young females. Most of the patients with MTS go through their life without having any symptoms. Its presentations range from being asymptomatic to severe un-resolving DVT. It should be suspected and investigated in patients with DVT at a young age not responding to conventional treatments. In a patient with unilateral leg swelling with a negative initial duplex ultrasound or non-resolving DVT, we should consider CT-venography to investigate a possible diagnosis of MTS [4].

In this patient, IV drug abuse is considered a predisposing factor leading to the development of DVT. MTS is often undiagnosed until an aggravating factor causes the de-compensation that is required to elicit the symptoms, such as during pregnancy or other prothrombotic states. DVT is diagnosed by a Doppler ultrasound. A high index of suspicion is generally required while investigating the cause of DVT. In the case of suspicion of DVT, venous Doppler ultrasound is the investigation of choice initially to look for filling defects [5].

When a patient presents with deep leg pain, swelling, or fullness in the background of predisposing factors such as pregnancy, IV-drug abuse, or any other prothrombotic state, a venous Doppler scan is initially ordered. CT-venogram is also done for a detailed overview of anatomical causes and to rule out thrombosis. Patients presenting with suspected MTS are thoroughly investigated for the types of thrombophilias (to look for all the possible causes leading to thrombosis). MTS has a few treatment modalities which include catheter-guided t-PA on the site of thrombosis, which causes immediate thrombolysis. An IVC filter is placed to prevent embolism, along with a metal stent at the site of left iliac vein compression from the right iliac artery to prevent the narrowing. This is then followed by long-term anticoagulation via direct oral anticoagulants like apixaban or rivaroxaban along with compression stockings. Rivaroxaban has been safe and effective in the treatment of various types of venous thromboembolism [6]. This is standard of care, there have been many instances where long-term filters can cause erosion into the vena cava and further complications. So, IVC filters are only placed temporarily in these cases and are removed within three months. Researchers have found that endovascular management of MTS or iliac vein compression has lesser recurrences and better outcomes as compared to those who were only managed by anti-coagulation or thrombectomy [7].

In a systematic review published by Kaltenmeier et al., it was reported that MTS is twice as common and is more likely to present with pulmonary embolism among females as compared to men, whereas men have had more pain and swelling as compared to women [8]. The most common treatment option noticed among 22 adolescent patients with MTS in a study conducted by Hansrani et al. was a combination of catheter-directed thrombolysis and iliac vein stenting (41%) compared to pharmaco-mechanical thrombolysis and iliac vein stenting (18%) [9].

Proximal vein thrombosis, if not treated early, has potentially hazardous consequences involving pulmonary embolism. So, patients should begin on immediate anticoagulation via low molecular weight heparin as compared to unfractionated heparin to lower the risk of heparin-induced thrombocytopenia. Afterward, a shift to vitamin K antagonists is usually recommended. Factor Xa inhibitors have been now proven to cause regression of massive deep vein thrombosis, along with having a lesser chance of recurrence and bleeding risk [10,11]. Catheter-directed thrombolysis is associated with a lesser risk of post-thrombotic syndrome as compared to conventional anticoagulation treatments to treat iliofemoral vein thrombosis [12]. Pharmacomechanical catheter-directed thrombolysis is known to remove iliofemoral thrombosis, lower residual thrombus after the procedure is associated with lesser severity of the post-thrombotic syndrome, improved venous quality of life, and fewer early symptoms [13]. A long-term follow-up of endovascular stenting for MTS showed good initial patency rates and symptomatic relief. It is for this reason that endovascular stenting was usually chosen as the main treatment for our patients. ​​Intravenous ultrasonography is a unique methodology that uses a specially designed catheter with a miniaturized ultrasound probe attached to the distal end of the catheter. Intravascular ultrasound (IVUS) can give us the exact internal surface area inside the vessel and also assist in appropriately quantifying the exact severity of stenosis and help in assessing the length and diameter of stent selection. For catheter-based procedures to prevent or treat VTE-related disorders, IVUS remains an essential tool for accurately imaging the major axial veins [14]. Due to the non-availability of the testing modalities, IVUS could not be performed on our patient.

## Conclusions

It is important to consider MTS as a differential diagnosis, especially in middle-aged patients with unilateral DVT. A timely diagnosis through relevant investigations is important due to the progressive nature of the disease and long-term disabling complications including post-thrombotic syndrome, venacaval erosions into surrounding viscera due to IVC filter, and pulmonary embolism. IVUS is important for determining the luminal diameter, the extent of occlusion, and the appropriate size and type of stent to be used. When encountering IV drug abuser patients, physicians need to maintain high clinical suspicion. The mainstay treatment remains to be the placement of a retrievable IVC filter, catheter-guided thrombolysis followed by angioplasty, and stenting of the iliac vein.
